# Left main coronary artery atresia in a 2-year-old toddler with *de novo* heart failure: Case report and review of the literature

**DOI:** 10.3389/fcvm.2022.898467

**Published:** 2022-10-21

**Authors:** Sahar Asl Fallah, Mohammad Mahdavi, Kiara Rezaei-Kalantari, Salah D. Qanadli, Saeed Mirsadraee

**Affiliations:** ^1^Tehran Heart Center, Tehran University of Medical Sciences, Tehran, Iran; ^2^Rajaie Cardiovascular Medical and Research Center, Iran University of Medical Sciences, Tehran, Iran; ^3^Cardiothoracic and Vascular Division, Department of Diagnostic and Interventional Radiology, Lausanne University Hospital and University of Lausanne, Lausanne, Switzerland; ^4^Department of Radiology, Royal Bromton Hospital, London, United Kingdom; ^5^National Heart and Lung Institute, Imperial College London, London, United Kingdom

**Keywords:** cardiac magnetic resonance (CMR), *de novo* heart failure, congenital coronary anomaly, left main artery atresia, anomalous left coronary artery from the pulmonary artery (ALCAPA)

## Abstract

Congenital coronary anomalies are among the rare disorders of the otherwise normal heart. A 2-year-old toddler was evaluated for *de novo* heart failure after a flu-like event 2 months before being suspicious of post-Covid-19 dilated cardiomyopathy. The cardiac magnetic resonance (CMR) technique displayed the basal to mid subendocardial to transmural scar, suggestive of an ischemic etiology. Further assessment with CT and invasive angiography confirmed the very uncommon left main coronary artery atresia (LMCAA) as the main cause of the patient's heart failure. This is not only the first reported LMCAA case that had undergone a CMR study but was also initially suspected with characteristic CMR findings.

## Introduction

Congenital coronary anomalies are rare disorders with a reported prevalence of 0.6–1.3% in angiographic series (1). Left main coronary artery atresia (LMCAA) is one of the least prevalent anomalies where the ostium of the left main (LM) artery is absent and the left anterior descending artery (LAD) and the left circumflex artery (LCX) connect blindly without direct origin from other vessels or cardiac chambers (2). While CT angiography (CTA) is the preferred method for the anatomic evaluation of coronary arteries (3), cardiac magnetic resonance (CMR) also plays an important role in myocardial tissue characterization for treatment strategy. We reported a case of LMCAA referred for CMR due to recent symptoms of heart failure. Written informed consent was obtained from the father of the patient for the publication of any potentially identifiable images or data included in this article.

## Case report

**Figure 1:**
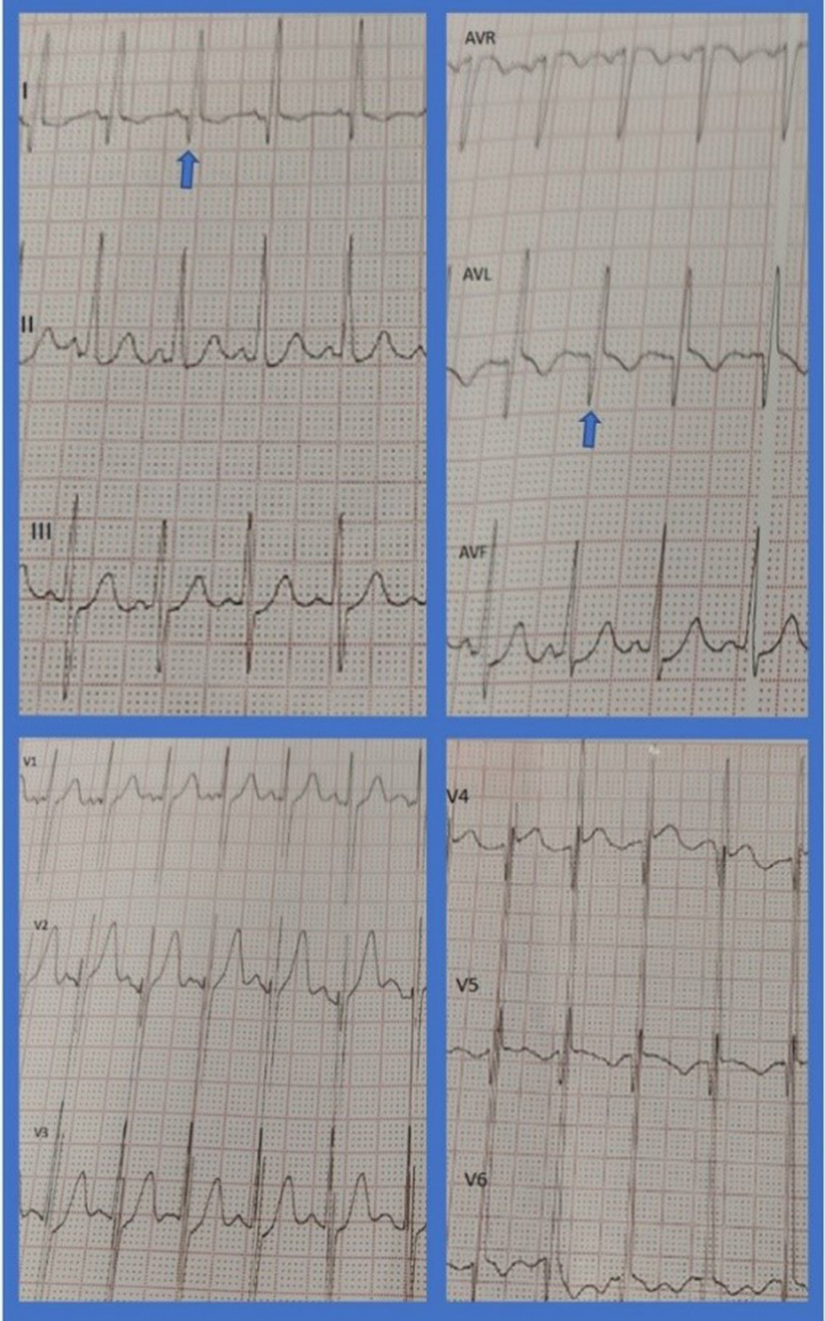
ECG demonstrating sinus tachycardia, pathologic Q waves in leads I, AVL, and V4–V6.

A 2-year-old toddler presented to a clinic with recent onset of abdominal pain, irritation, and failure to thrive. The patient had experienced flu-like symptoms 2 months before being suspicious of the COVID-19 infection. Outpatient workup revealed significantly dilated left ventricle (LV) and impaired systolic LV function with an EF of 20–25% and severe functional mitral regurgitation (MR). On physical examination, S1, S2, and S3 were audible with an early systolic grade 4 murmur. The patient's lungs were clear. Electrocardiography showed sinus tachycardia (150 bpm) and deep Q waves in leads I, AVL, V4, V5, and V6 in favor of chronic LV myocardial injury (Figure 1). Cardiac troponin, ESR, and CRP levels were normal. The ProBNP level increased (1,950 pg/ml) and COVID-19 PCR was tested negative.

**Figure 2:**
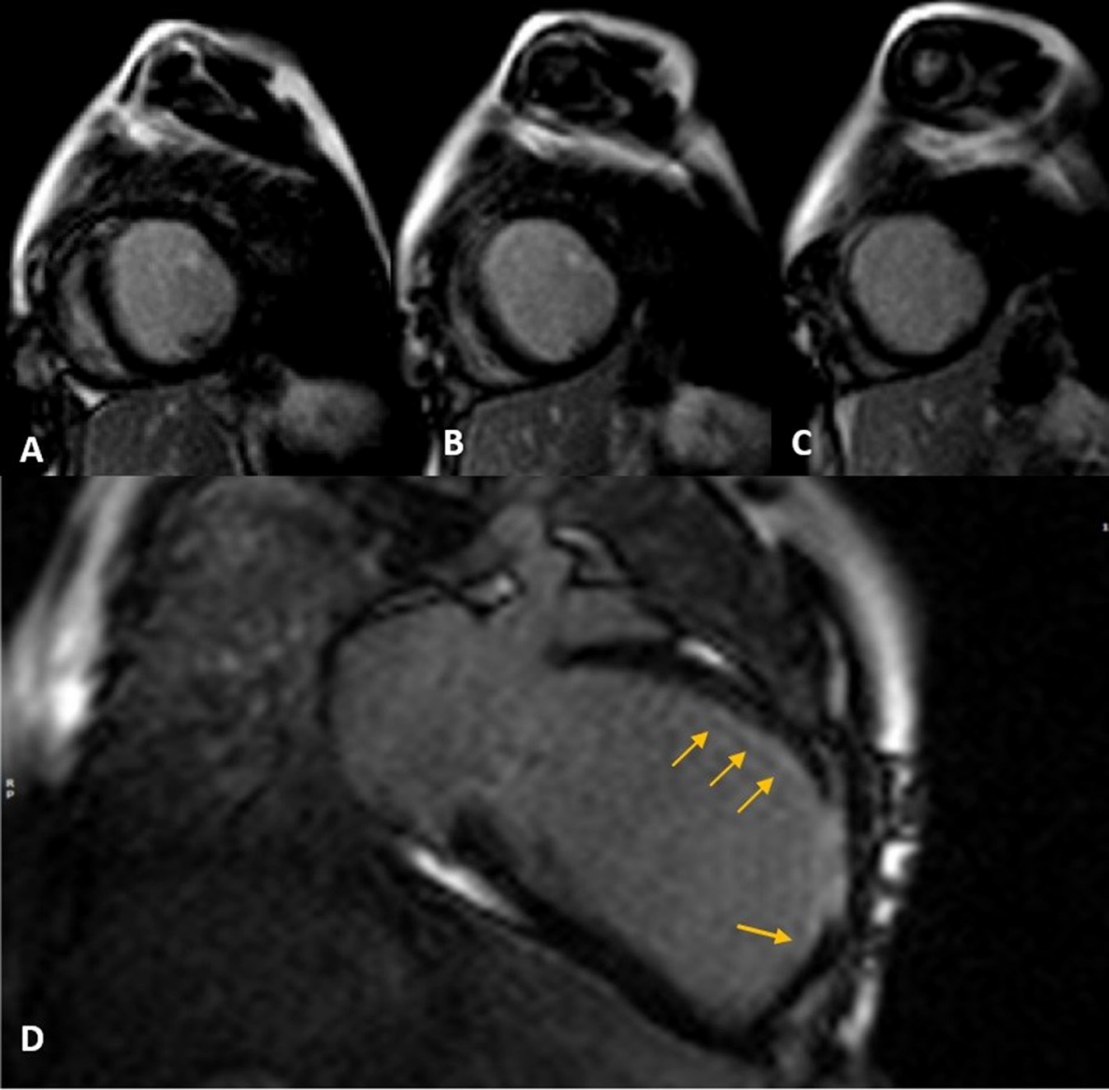
Late gadolinium enhancement (LGE) sequence images in short **(A–C)** and long **(D)** axis views show subendocardial (thin arrows) to transmural scar concentrated in mid-anteroseptal and anterior LV segments.

**Figure 3:**
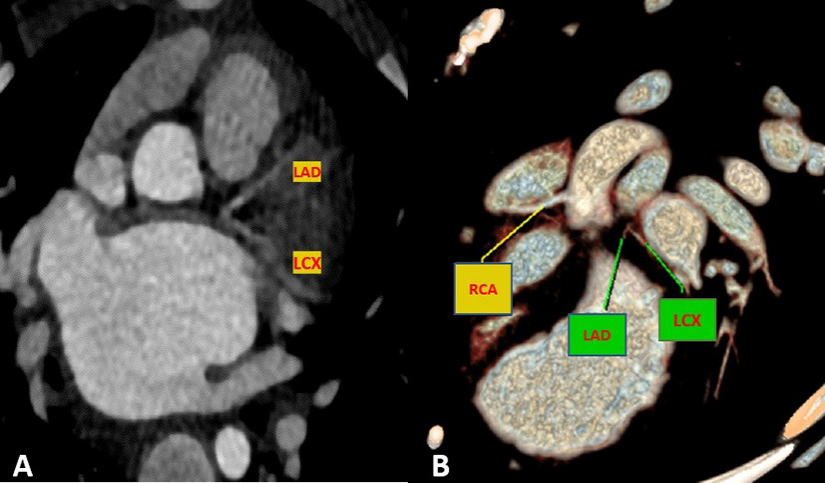
Maximum intensity projection **(A)** and volume rendered **(B)** CT images demonstrate an absence of the LM artery with proximally connected left anterior descending artery (LAD) and left circumflex artery (LCX).

**Figure 4:**
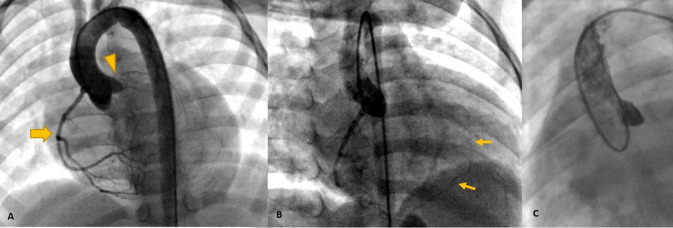
Invasive angiography **(A–C)** showing the dominant RCA [Thick arrow in **(A)**] retrogradely filling the left system *via* collaterals [Thin arrows in **(B)**] and no antegrade flow in left cusp injection **(C)** confirming left main atresia (arrowhead).

Cardiac magnetic resonance was performed to investigate the possible underlying pathology, which confirmed severe LV dilation, severely reduced systolic function (LVEF: 22%), and severe MR (Figure 2). Late gadolinium enhancement (LGE) sequence demonstrated subendocardial to transmural scar in the basal and especially at the mid anterior and anteroseptal segments, raising suspicion of an ischemic insult. Gated CT coronary angiography (Figure 3) demonstrated an absent LM stem with a centripetal filling of the small caliber confluent LAD and circumflex, likely from collaterals from a dilated right coronary artery. Angiography confirmed the LM atresia with a retrograde collateral filling of LAD and LCX arteries from a dominant RCA. There was no connection to the pulmonary arterial branches (Figure 4; Supplementary Videos 1, 2). Considering the extent of myocardial fibrosis, small-sized coronary arteries and LV remodeling were required, and according to the decision of heart team specialists, the patient underwent medical treatment for heart failure and was scheduled for a heart transplant.

## Discussion

To our knowledge, this is the first LMCAA case suspected initially from the findings of CMR of characteristic ischemic LGE pattern and further confirmed by the CTA and invasive angiography.

The left main coronary artery atresia is one of the rarest congenital coronary anomalies with <100 reported cases, in which an enlarged RCA is responsible to provide perfusion for the left system *via* collaterals. The clinical presentation depends mostly on the capacity and size of collateral arteries and the site of their connection to the left system and is variable from asymptomatic to congestive heart failure, syncope, and sudden cardiac death (SCD) (2, 4). Small and distally connected collaterals are indicative of poor outcomes compared to larger, more prominent, and proximally connected ones (1).

A case series study with a review of the literature, which was published in 2019, analyzed previously reported cases of 50 pediatric patients and 45 adult patients with LMCAA. In total, 88% of the pediatric group was symptomatic while 79% of the adult group was symptomatic. Heart failure was reported to be the most common finding in pediatric patients occurring in 44%, followed by syncope (28%). In the adult group, angina was the most prevalent symptom (48.8%) followed by exertional dyspnea (14%). Sudden cardiac death was reported in 10 and 7% of the pediatric and adult groups, respectively (2).

The coronary artery anatomy and anomalous origins are best visualized with multislice CTA due to its high spatial resolution and reconstruction techniques (3). Invasive angiography is used to help confirm the diagnosis (1, 2).

Differentiating LMCAA from other conflicting diagnoses such as absent LM with a single coronary artery, an anomalous left coronary artery from the pulmonary artery (ALCAPA), and secondary occlusion of LM coronary artery is crucial (Supplementary Table 1).

The left main coronary artery atresia is completely different from absent LM with a single coronary artery, which occurs in <1% of congenital coronary anomalies (5). It is a condition where a single ostium coronary artery from the aortic trunk may have either RCA or LM origin and divides proximally to take a normal RCA/LCA course or a totally different coronary tree with the antegrade flow (6). This is mostly taken as a normal variation since it rarely causes symptoms (7). In both LMCAA and single coronary with right coronary cusp origin, RCA is dilated; however, in LMCAA, the blood supply to the left system is retrogradely perfused by collaterals (1).

Another common misdiagnosis for LMCAA is ALCAPA occurring in 1 in 300,000 live births (3). A total of 26% of pediatric patients reported in Alsalehi et al.'s (2) review were first misdiagnosed with ALCAPA. In both ALCAPA and LMCAA, RCA is dilated; however, in ALCAPA, the proximal part of the left system finally drains to the pulmonary artery. An accurate diagnosis of LMCAA from ALCAPAS is important for preoperative planning and pulmonary artery division (2, 6).

An anomalous left coronary artery from the pulmonary artery causes chronic ongoing myocardial hypoperfusion leading to myocardial hibernation, which dramatically improves after coronary reimplantation (6, 8). CMR has the advantage of distinguishing hibernated from irreversibly infarcted myocardium (9). In a case series of patients with ALCAPA undergoing CMR, only 25% of patients with ALCAPA showed scar in their LGE sequence, which validated the fact that myocardium is mainly viable despite depressed ventricular function (8). Nonetheless, no CMR or clinical data in patients with LMCAA on myocardial viability or functional reversibility after revascularization have been reported to this date. Regarding the retrograde flow dynamic pattern of coronary arteries in both LMCAA and ALCAPA, the ischemic scar might have basal/mid LV segment predilection due to more downstream locations in the retrograde perfusion pathway. However, further investigation is required for a thorough assessment.

Total occlusion of the LM has a relatively similar manifestation to LMCAA as demonstrated in reported cases of arteritis by Takayasu (10) and Kawasaki (11); Cross-sectional imaging often reveals the occluded portion, sometimes accompanied by adjacent aortic wall thickening.

Patients with LMCAA should undergo surgical correction with the restoration of the antegrade flow to the left coronary system either by osteoplasty or bypass grafting. Otherwise, the condition deteriorates gradually with a poor outcome. In instances in which reperfusion is not possible (due to small coronary artery diameter) or not effective (due to extensive transmural myocardial scar), the treatment strategy will focus on heart failure management till the provision of condition for heart transplantation as the last remaining option.

## Data availability statement

The original contributions presented in the study are included in the article/Supplementary material, further inquiries can be directed to the corresponding author.

## Ethics statement

Ethical review and approval was not required for the study on human participants in accordance with the local legislation and institutional requirements. Written informed consent to participate in this study was provided by the participants' legal guardian/next of kin. Written informed consent was obtained from patient father for the publication of any potentially identifiable images or data included in this article.

## Author contributions

SA, KR-K, and MM contributed to gathering data and writing the original draft. SQ and SM contributed to editing and reviewing the manuscript. All authors contributed to the article and approved the submitted version.

## Conflict of interest

The authors declare that the research was conducted in the absence of any commercial or financial relationships that could be construed as a potential conflict of interest.

## Publisher's note

All claims expressed in this article are solely those of the authors and do not necessarily represent those of their affiliated organizations, or those of the publisher, the editors and the reviewers. Any product that may be evaluated in this article, or claim that may be made by its manufacturer, is not guaranteed or endorsed by the publisher.
