# Supplementary material for: Left main coronary artery atresia in a 2-year-old toddler with de novo heart failure: Case report and review of the literature
Source: Front Cardiovasc Med. 2022 Oct 21;9:898467. doi: 10.3389/fcvm.2022.898467 (PMC9634233; doi:10.3389/fcvm.2022.898467)
Supplement: Supplementary file 1 [file Table_1.docx]

|  | **LM** | **RCA** | **Left coronary perfusion** | **Left coronary tree anatomy** |
| --- | --- | --- | --- | --- |
| **LMCAA** | Atresia of LM ostium | RCA is the dominant coronary artery | Retrograde perfusion via collateral arteries from RCA | Small sized proximal portion with more prominent distal branches |
| **ALCAPA** | LM ostium connected to the pulmonary artery | RCA is the dominant coronary artery | Retrograde perfusion via collateral arteries from RCA finally draining to pulmonary artery | Prominent proximal portion relative to small sized distal branches |
| **Single coronary artery with RCA origin** | Absent LM | RCA is the dominant coronary artery | Antegrade perfusion via collateral arteries from RCA | variable |
| **Chronic Total occlusion of LM** | Presence of LM trunk with totally occluded lumen | RCA is the dominant coronary artery | Retrograde perfusion via collateral arteries from RCA | Normal but poor flow left coronary tree |

STable 1: Differential diagnosis for left main artery atresia.
